# The sexually dimorphic impact of maltreatment on cortical thickness, surface area and gyrification

**DOI:** 10.1007/s00702-016-1523-8

**Published:** 2016-02-27

**Authors:** Philip A. Kelly, Essi Viding, Vanessa B. Puetz, Amy L. Palmer, Sophie Samuel, Eamon J. McCrory

**Affiliations:** 1 Division of Psychology and Language Sciences, University College London, Gower Street, London, WC1 6BT UK; 2 The Anna Freud Centre, Maresfield Gardens, London, NW3 5SU UK

**Keywords:** Maltreatment, Sex differences, Cortical thickness, Gyrification, Surface area, Latent vulnerability

## Abstract

An extensive literature has detailed how maltreatment experience impacts brain structure in children and adolescents. However, there is a dearth of studies on the influence of maltreatment on surface based indices, and to date no study has investigated how sex influences the impact of maltreatment on cortical thickness, surface area and local gyrification. We investigated sex differences in these measures of cortical structure in a large community sample of children aged 10–14 years (*n* = 122) comprising 62 children with verified maltreatment experience and 60 matched non-maltreated controls. The maltreated group relative to the controls presented with a pattern of decreased cortical thickness within a region of right anterior cingulate, orbitofrontal cortex and superior frontal gyrus; decreased surface area within the right inferior parietal cortex; and increased local gyrification within left superior parietal cortex. This atypical pattern of cortical structure was similar across males and females. An interaction between maltreatment exposure and sex was found only in local gyrification, within two clusters: the right tempo-parietal junction and the left precentral gyrus. These findings suggest that maltreatment impacts cortical structure in brain areas associated with emotional regulation and theory of mind, with few differences between the sexes.

**Electronic supplementary material:**

The online version of this article (doi:10.1007/s00702-016-1523-8) contains supplementary material, which is available to authorized users.

## Introduction

Childhood maltreatment is increasingly recognised as major public health concern (Gilbert et al. 2009). It significantly increases the risk of a range of psychiatric disorders across the lifespan (Gilbert et al. 2009), including depression, anxiety and post-traumatic stress disorder (Anda et al. 2006; De Bellis 2001; Scott et al. 2010). Early adolescence represents a critical time in development when many psychiatric disorders begin to emerge (Paus et al. 2008; Uhlhaas and Singer 2011) and many of these disorders are characterized by different patterns of prevalence, age of onset and symptomatology between males and females (e.g. Crijnen et al. 1997; Leadbeater et al. 1999). Sex differences have also been reported for psychiatric outcomes in maltreated samples. According to the extant literature, females are more likely to present with internalising and males with externalising symptomatology after maltreatment experience (Bos et al. 2011; Keyes et al. 2012). However, we know relatively little about neurobiological sex differences in individuals who have experienced maltreatment; furthermore, the research to date has focussed on volumetric differences (Lim et al. 2014). Characterizing the neurobiological sequelae of childhood maltreatment across males and females in relation to more fine-grained surface level indices may provide clues as to possible sex differences in patterns of latent vulnerability to subsequent psychiatric disorder (Kelly et al. 2015; McCrory and Viding 2015).

### The impact of maltreatment on gray matter volume and the influence of sex

A growing number of investigations have now associated maltreatment exposure with atypical gray matter volume (GMV) in child and adult samples. A recent meta-analysis that combined twelve studies of adults and children reported a broad pattern of reduced GMV in maltreated individuals in a number of regions, including the orbitofrontal gyrus, the superior and middle temporal gyri, amygdala, insula, and para-hippocampal gyri (Lim et al. 2014). However, given that this meta-analysis combined studies of child and adult samples, the majority of which were comprised of individuals with concurrent psychiatric disorders, one must be cautious in assuming specificity of all of these GMV differences to maltreatment experience in children (Lim et al. 2014).

To date, few studies have investigated the potential interaction between maltreatment and sex in relation to brain structure (De Bellis and Keshavan 2003; De Bellis et al. 1999; De Bellis and Kuchibhatla 2006; Edmiston et al. 2011). Structural sex differences have been noted in a number of regions that are also significantly reduced following maltreatment (Chen et al. 2007; Koolschijn and Crone 2013; Peper et al. 2009). A set of early influential studies by De Bellis and colleagues, found that maltreated boys with PTSD presented with smaller cerebral volumes and larger ventricular volumes in comparison to maltreated females with PTSD (De Bellis and Keshavan 2003; De Bellis et al. 1999), implying that maltreatment exposure may have a differential impact in males than females. One correlational study has explored sex differences and the impact of maltreatment on regional GMV in a single group of children exposed to adversity (Edmiston et al. 2011). Namely, Edmiston and colleagues found that early trauma was associated with GMV decreases in emotional regulation areas in females including dorsolateral and orbitofrontal cortex, and decreases in caudate regions in males. Unfortunately, it was not possible to ascertain that the regions associated with sex differences reflected sex differences in response to maltreatment, as opposed to potential confounds such as IQ or SES (which were not accounted for in this analysis).

In a recent volumetric study of sex differences in maltreated children, free of psychiatric diagnosis, we found that reduced GMV in the medial orbitofrontal cortex (mOFC), bilateral middle temporal lobes (MTL) and bilateral supramarginal gyrus in maltreated children compared to matched controls (Kelly et al. 2015). Sex differences were observed only in the supramarginal gyrus and the postcentral gyrus, but it appeared that maltreatment exerted largely similar effects on GMV in both sexes. However, measurement of GMV may in fact provide a relatively blunt index of altered brain structure and more subtle sex differences may be apparent when brain structure is interrogated by surface-based methods.

### The determinants of GMV

GMV is determined by two separable cortical indices, cortical thickness (CT) and surface area (SA), which are under distinct genetic influence and which have different developmental trajectories (Panizzon et al. 2009; Schaer et al. 2008). Local gyrification (lGI) is another surface-based property which reflects cortical complexity and folding, and is suggested to support increases in cortical surface area (Reillo et al. 2011). CT is a reflection of cortical layers (Rakic 1988), density of neurons and related developmental processes, such as myelination (Paus et al. 1999; Sowell 2004). SA, while also providing a reflection of neuronal density (Sisodiya and Free 1997; Sisodiya et al. 1996), reflects the number of cortical columns within each region (Rakic 1988). Structural variations within these cortical indices have been associated with psychiatric disorders, such as conduct disorder (Hyatt et al. 2012), schizophrenia (Nesvåg et al. 2014; Palaniyappan and Liddle 2012) and depression (Jaworska et al. 2014; Wagner et al. 2012). Variations in these indices can provide us with important clues about underlying neurodevelopment; for instance cortical thickness reductions are believed to optimise computation in frequently used circuits (Sowell et al. 2007), while alterations in cortical gyrification have been associated with neural connectivity patterns (Van Essen 1997).

### Sex differences, maltreatment and surface based indices

Consistent with studies indicating global and local GMV differences between males and females (Allen et al. 2003; Chen et al. 2007; Cosgrove et al. 2007; Koolschijn and Crone 2013; Luders et al. 2009; Peper et al. 2009; Shin et al. 2005), CT and SA and their developmental trajectory have been reported to differ between the sexes (Im et al. 2006; Luders et al. 2005, 2006; Lv et al. 2010; Mutlu et al. 2013; Raznahan et al. 2011; Sowell et al. 2007). It is also noteworthy that in relation to gyrification females tend to show patterns of highly localised increases compared to men (Luders et al. 2006).

A limited number of studies have investigated surface based measures in relation to maltreatment exposure. These previous studies discovered associations between early adversity and atypical cortical thickness, such as reductions in the lingual gyrus (Tomoda et al. 2012), the left anterior cingulate cortex (Heim et al. 2013) and accelerated thinning in frontal and precentral areas (Whittle et al. 2013). In our own study of cortical thickness, surface area and gyrification in children who had experienced documented maltreatment, we reported alterations in all surface based measures compared with carefully matched controls (Kelly et al. 2013). Specifically, reduced cortical thickness was observed in an extended cluster that incorporated the anterior cingulate, superior frontal gyrus and orbitofrontal cortex, with a number of regional differences in surface area and gyrification including reduced surface area in the middle temporal lobe and lingual gyrus. However, because the sample was relatively small, it was not possible to investigate sex differences, therefore we do not know whether the findings were equally driven by the males and females within the sample. To our knowledge, no study to date has investigated the sexually dimorphic impact of maltreatment on surface-based cortical indices.

#### The present study

Our primary aim was to investigate regional differences in cortical thickness, surface area and gyrification associated with maltreatment and to subsequently investigate possible sex differences in how maltreatment may impact these indices. Given that surface area is thought to show more sexual dimorphism than cortical thickness (Raznahan et al. 2011), we expected that sex differences following maltreatment exposure might be particularly apparent in relation to surface area and its determinant, local gyrification. We predicted that the general pattern of maltreatment related differences would overlap with those of our previous surface-based study (Kelly et al. 2013).

## Method

### Participants

A total of 122 children aged 10–14 years were recruited from London and the South-East of England as part of two related studies investigating the neural correlates of childhood maltreatment. Children with documented experiences of maltreatment (physical, sexual, emotional abuse or neglect; total *n* = 62) were recruited from Social Services (SS) departments in London (*n* = 52) and affiliated adoption agencies (*n* = 10). An additional *n* = 60 comparison children who had no documented history of maltreatment were recruited from primary and secondary schools, as well as after-school youth clubs in the London area, and via newspaper and internet advertisement. Exclusion criteria for the comparison group included any previous contact with SS with regard to the quality of care or maltreatment of the child. Participants in the maltreated and comparison groups were matched on age, pubertal status, sex, handedness, cognitive ability, socio-economic status and ethnicity (see Table 1).

**Table 1:** Socio-demographic characteristics and psychiatric symptomatology for the maltreated and non-maltreated groups

	Control (*n* = 60)	MT (*n* = 62)	*p*
Socio-demographic measures			
Tanner stage			.61
Pre/early pubertal (%)	15 (25)	22 (35)	
Mid pubertal (%)	23 (38)	24 (39)	
Late/post pubertal (%)	22 (37)	16 (26)	
Sex, *n* of males (%)	25 (42)	33 (53)	.20
Ethnicity, *n* of Caucasian (%)	31 (52)	39 (63)	.21
Handedness, *n* of right handed (%)	53 (88)	46 (74)	.37

	Mean	SD	Mean	SD	*p*
Age (years)	12.68	1.14	12.24	1.52	.07
WASI, 2 scale subset^a^	108.88	10.49	104.81	13.23	.06
Puberty Development Scale	2.22	.66	2.04	.71	.15
SES composite score	3.15	.87	2.89	1.08	.22
Psychiatric symptomatology					
TSCC					
Anxiety	45.26	11.17	45.72	15.95	.87
Depression	44.19	9.94	44.08	14.55	.97
Anger	41.84	9.07	43.80	13.35	.42
Post-traumatic stress	43.02	8.30	45.60	14.48	.31
Dissociation	43.28	11.46	45.88	13.92	.33
Dissociation (overt)	45.28	9.52	45.78	13.68	.84
Dissociation (fantasy)	44.33	11.28	46.90	14.63	.35
SDQ					
Emotional symptoms	2.38	2.05	3.22	2.65	.06
Conduct problems	1.32	1.42	2.70	2.05	.00
Hyperactivity/inattention	2.68	1.99	4.23	2.65	.00
Peer problems	1.22	1.27	2.03	1.90	.01
Total difficulties	7.46	5.05	11.48	7.42	.01

All *p* values derived from *t* tests with the exception of sex, ethnicity, handedness and Tanner stage comparisons which used Chi square tests

*SES* socio-economic status, *TSCC* Trauma Symptom Checklist for Children, *SDQ* Strengths and Difficulties Questionnaire

^a^No participant scored below 70 or above 130 on the WASI

Assent to participate in the study was obtained for all children. For children living with their biological or adoptive parents, consent was obtained from at least one parent. Where there was shared parental responsibility with SS, consent was obtained from the biological parent of the child (if contactable), and SS. Exclusion criteria for all participants included a diagnosis of learning disability, pervasive developmental disorder, neurological abnormalities, standard MRI contra-indications (e.g. ferromagnetic implants or braces) and cognitive ability (WASI) <70. All procedures in the study were approved by University College London Research Ethics Committee (0895/002).

A subset of participants included in the present study (43/122; 22 maltreated and 21 non-maltreated) had been included in a previous study examining the impact of maltreatment on surface-based indices of cortical structure (Kelly et al. 2013). The same magnetization prepared rapid gradient echo sequence was employed on the identical MRI scanner for all participants. The present sample is identical to that included in a recent study of maltreatment, sex differences, GMV and attentional bias (Kelly et al. 2015).

### Measures

#### Maltreatment history

Social services case files for the maltreated group were independently rated on a child-maltreatment rating scale (Kaufman et al. 1994). This five point scale is rated from 0 = ‘no abuse present’ to 4 = ‘evidence of severe abuse’ by the child’s social worker or the adoptive parent based on information provided by social services in relation to each maltreatment subtype. As is typically found, most maltreated individuals experienced more than one form of maltreatment (90.30 % of the sample were reported to have experienced two or more forms). The most commonly reported forms of maltreatment were neglect (82.26 % of sample here; *M* = 3.24, SD = 1.12) and emotional abuse (93.55 % of sample; *M* = 2.93, SD = .92). Physical abuse (19.35 %, *M* = 1.92, SD = 1.16) and sexual abuse (12.90 % here, *M* = 2.29, SD = 1.38) were less common.

#### Cognitive ability

Participants were administered the vocabulary and matrix reasoning subtests of the Wechsler Abbreviated Scale of Intelligence (WASI; Wechsler 1999) in order to provide an estimated Full Scale Intelligence Quotient (FSIQ).

#### Socio-economic status

Current socio-economic status was assessed using information collected from the parent or caregiver, including highest level of education, household income, and current occupation. Highest level education was rated on a 6 point scale from 0 =‘no formal qualifications’ to 5 =‘postgraduate qualification’. Household income was rated on an 8 point scale from 1 = ‘£0–£10,000’ to 8=‘£60,000–£70,000+’. Current occupation of the primary care giver was classified using the National Statistics Socio-economic Classification’s Standard Occupation Classification 2000 manual (Office for National Statistics 2005) on a four class scale from 1 =‘managerial and professional occupation’ to 3 = ‘routine and manual occupation’ with four coding for participants who had never worked or were long-term unemployed. The measure of occupation was reverse coded and a composite score was derived from the mean of these three scales, so that a greater score indicated a greater level of socio-economic status.

#### Pubertal status

Pubertal development was assessed with both the self-report and parent-rated eight-item Puberty Development Scale (PDS; Petersen et al. 1988). An average pubertal development scale and a two stage indicator of pubertal development based upon Tanner stage were derived from these scores. There was a 72.3 % agreement between parent and child reported two-level indicator of pubertal development.

#### Psychiatric symptomatology

The Trauma Symptom Checklist for Children (TSCC; Briere 1996) was used to assess posttraumatic symptomatology and other symptom clusters. This 44-item self-report measure has five clinical scales (anger, depression, anxiety, posttraumatic stress and dissociation) and two validity scales (under- and hyper-response). Each item is rated on a four-point scale from ‘never’ to ‘almost all the time’. Cronbach’s alpha for the scales varied from .84 to .88.

The Strength and Difficulties Questionnaire (SDQ; Goodman 1997), a 25 item self-report measure was included to assess general psychological and behavioural functioning. The SDQ included five behavioural scales (emotional symptoms, conduct problems, hyperactivity, peer problems, and prosocial behaviour) and a total difficulties score. Items were rated from ‘not true’ to ‘certainly true’ on a three-point scale. Cronbach’s alpha for the scales varied from .65 to .81.

### MRI acquisition

Participants were scanned with a 1.5 T Siemens (Siemens Medical Systems, Munich, Germany) Avanto MRI scanner with a 32-channel head coil. A high-resolution, three-dimensional T1-weighted structural scan was acquired with a magnetization prepared rapid gradient echo sequence. Imaging parameters were: 176 slices; slice thickness = 1 mm; gap between slices = .5 mm; echo time = 2730 ms; repetition time = 3.57 ms; field of view = 256 mm × 256 mm^2^; matrix size = 256 × 256; voxel size = 1 × 1 × 1 mm resolution. The scanning time was 5.5 min. Foam padding was used against the sides and the back of the head of the participant, to minimize head motion. Ear buds attenuated scanner noise.

### MRI processing and analysis

All T1-weighted images were initially manually inspected for any deformations or inconsistencies that may impede its processing such as movement artefacts or structural abnormalities. If the image was thought to represent poor quality or had any ostensible deformations, the participant was excluded. From an initial recruitment of 137 participants, a total of 15 participants (MT = 8, non-MT = 7) were excluded from the analysis due to concerns over image quality, resulting in a final sample of 122 (Table 1).

Cortical reconstruction was performed with the FreeSurfer image analysis suite (Dale et al. 1999; Fischl and Dale 2000; Fischl et al. 1999a, 2004). In brief, the initial steps in this well-validated (Dale et al. 1999; Fischl et al. 1999a; Ségonne et al. 2004) surface based morphometric pipeline were as follows. Removal of non-brain tissue using a hybrid watershed/surface deformation procedure (Ségonne et al. 2004), automated Talairach transformation, intensity normalization (Sled et al. 1998), tessellation of the gray matter white matter boundary, automated topology correction (Fischl et al. 2001; Ségonne et al. 2007), and surface deformation following intensity gradients to optimally place the gray/white and gray/cerebrospinal fluid borders at the location where the greatest shift in intensity defines the transition to the other tissue class (Dale et al. 1999; Dale and Sereno 1993; Fischl and Dale 2000). The structural measures were calculated in native space of each participant and transformed into a spherical representation which are registered to a common spherical atlas. The atlas is based on individual cortical folding patterns to match cortical geometry across subjects, preserving the vertex identities (Fischl et al. 1999b). All participant’s surface models were inspected for accuracy and manual edits were made when there were inconsistencies in the differentiation between pial surface and other organic matter, including dura and bone.

Cortical thickness at each vertex was measured by calculating the shortest distance from the gray/white matter boundary to the pial surface (in millimetres). Surface area measurement was quantified by assigning an area to each vertex equal to the average of its surrounding triangles. When the vertex areas are summed over all vertices, the total is equal to the sum of the areas of the triangles. The surface area was calculated at the pial level and represents the area of vertex on the gray matter surface, calculated as the average of the area of the tessellated triangles touching that vertex. Parcellation of each participant’s cortex into gyral regions was based on the Desiken-Killiany atlas (Desikan et al. 2006). Surface area was not analysed at the vertex-level as the values provided by the standard FreeSurfer pipeline do not provide an accurate measure of surface area, rather it indicates a measure of areal expansion to the study template. The average surface area value for each parcellated region provides an accurate measure of surface are and was extracted for all participants.

The local gyrification index (lGI) is a supplementary measure incorporated within the FreeSurfer image analysis suite which takes into account the intrinsic 3D nature compared with 2D methods (Schaer et al. 2008). The lGI method uses the pial and white matter surface identification against an additional outer hull layer that tightly wraps the pial surface. The lGI value at each vertex is computed within 25 mm circular regions of interest and represents the ratio of pial surface to outer hull surface, an indication of sulcal cortex buried in its locality and thus the extent of cortical folding. See Schaer and colleagues (Schaer et al. 2008) for further details of this analytic approach. Vertex-level cortical thickness, and local gyrification index for each participant were mapped onto a normalised cortical surface to perform group analysis and to investigate the interaction between sex and maltreatment exposure. Analyses on extracted cortical surface area values were performed within SPSS v.20 (IBM, Armonk, NY).

### Statistical analysis

#### Group differences

Regionally specific between group differences in cortical thickness, surface area and lGI were investigated within the QDEC application of FreeSurfer using a two sample *t* test model. Cortical thickness measurements were smoothed with a full-width-at-half-maximum kernel of 15 mm. Local gyrification index measurements were not smoothed due to lGI maps being inherently smooth (given that GI is calculated in a radius of 25 mm). Excessive smoothing of the lGI data can contribute to the failure in computing Monte-Carlo null-*z* simulation to correct for multiple comparisons. Between group differences were corrected for multiple comparisons with a Monte Carlo simulation (*p* < .05 two-tailed; to maximise sensitivity to potential group differences and sex by group interactions) and adjusted for interhemispheric comparisons. When regions of difference were found to be significant between the maltreated and non-maltreated groups, the mean structural value was extracted from the significant cluster in participant’s native space. Cortical thickness, surface area, and local gyrification undergo dynamic changes during childhood and adolescence and are known to be influenced by IQ and age (Giedd and Rapoport 2010; Raznahan et al. 2011; Shaw et al. 2006). Although there were no significant group differences in age, sex, and IQ, our statistical models were run with these variables and intracranial volume (ICV) included as covariates of no interest. These extracted cortical thickness, surface area and local gyrification values were imported into SPSS v.20 (IBM, Armonk, NY) to undertake further analysis to examine the relationship with maltreatment characteristics and psychiatric symptomatology, as well as a potentially confounding influence of pubertal stage. A mixed-model analysis of variance (ANOVA) was used to assess group differences in gyral level surface area across the whole cortex. Group (maltreated versus non-maltreated) was assigned as the between subject factor and region (34 gyral regions per hemisphere) as the within-subject factors. Independent *t* tests were used to examine group differences in gyral level cortical thickness. Due to the relatively large number of independent *t* tests, a false discovery-rate correction was performed to control for multiple comparisons across both hemispheres (Benjamini and Hochberg 1995).

#### Sex differences

To investigate sex differences in the impact of maltreatment on surface-based cortical indices, two statistical approaches were implemented. First to investigate whether the local significant differences in the surface-based measures associated with maltreatment were driven primarily by an effect in males and females, a 2 × 2 ANOVA was implemented within SPSS on the extracted cortical thickness, surface area and local gyrification values. Second, to investigate group by sex interactions at the level of the whole brain, which may have been occluded in the main effect of group analysis, sex and group were included as variables of interest in QDEC and an interaction on a whole brain scale (by hemisphere) was examined. Again, age, IQ, and ICV were included as covariates of no interest within all statistical models. In an additional step pubertal status was included as a covariate of no interest. Potential interactions between sex and maltreatment exposure in surface area were examined by parcellated gyral region within SPSS, using a similar model design implemented in FreeSurfer. A mixed-model analysis of variance (ANOVA) with group and sex as between-subject factors and gyral region as within-subject factors was used, and corrected for multiple comparisons (Benjamini and Hochberg 1995).

## Results

### Socio-demographic variables, psychiatric symptomatology and global cortical measures

The maltreated group did not differ from the non-maltreated group in relation to sex, age, pubertal status, handedness, IQ, SES and ethnicity (Table 1). The maltreated group relative to the non-maltreated group did not differ on any of the subscales of the TSCC, but did show heightened scores on the conduct problems [*t*(_122_) = −4.25, *p* < .001] and hyperactivity [*t*(_122_) = −3.62, *p* < .001] subscales of the SDQ. There were no significant interactions between sex and maltreatment exposure on any of the subscales of the SDQ and the TSCC. The maltreated and non-maltreated groups did not differ on intracranial volume (ICV), total surface area or mean cortical thickness and lGI values, and group by sex interactions were not significant for any of these cortical measures. However, surface area and estimated ICV were found to be greater in males compared to females across both groups (Supplemental Table S1.)

### Group differences

#### Cortical thickness

**Fig. 1:**
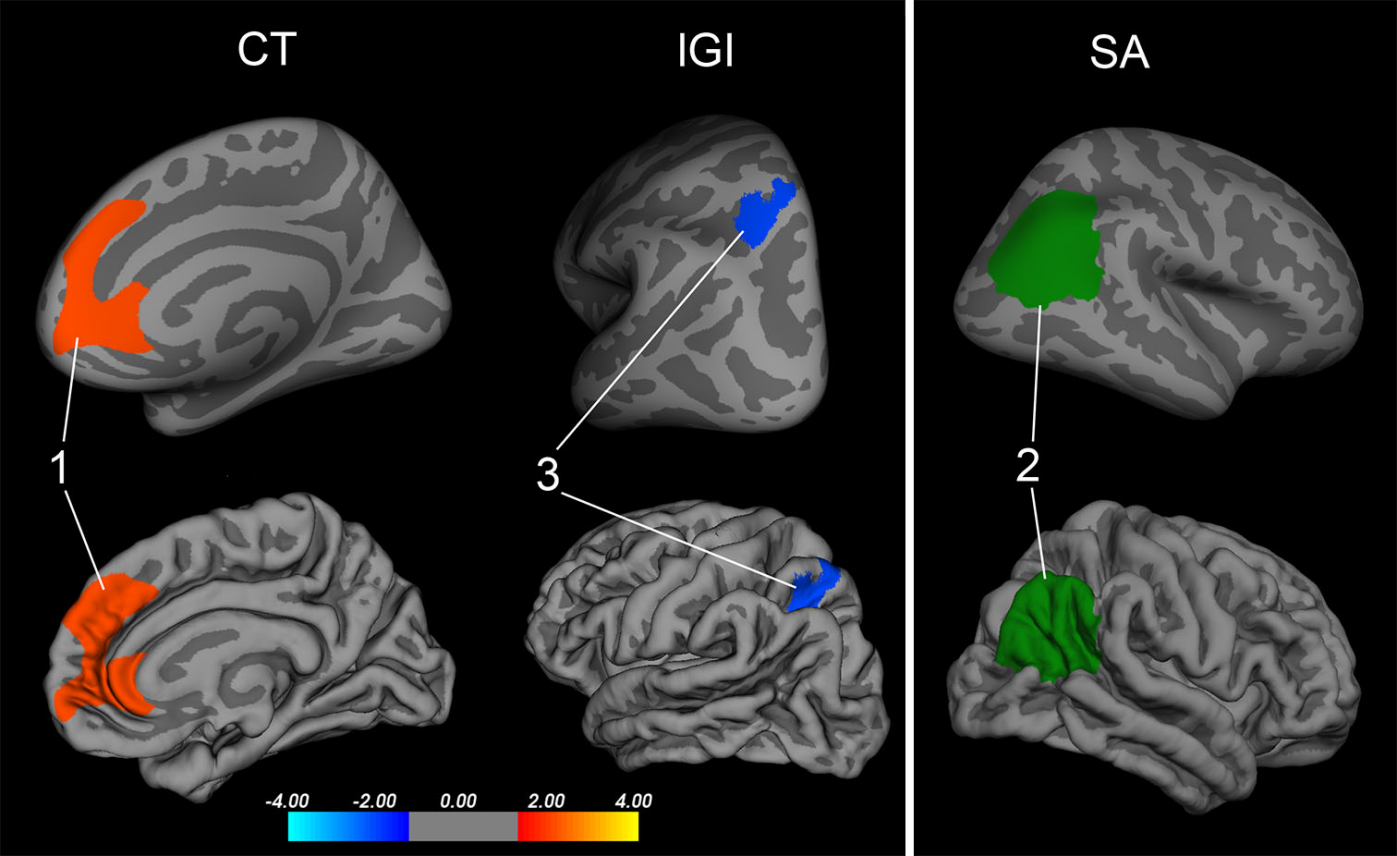
Clusters of significantly different cortical structure between the maltreated group and the non-maltreated peers. Cluster *1* indicates a significantly reduced right hemisphere region of cortical thickness (CT) among the maltreated children relative to the controls within a region that encompasses the orbitofrontal cortex, superior frontal gyrus and anterior cingulate cortex. Cluster *3* indicates a significant left hemisphere increase in local gyrification index (lGI) in a region of the superior parietal cortex within the maltreated children compared to the controls. Cluster *2* depicts the parcellated gyral region found to have decreased surface area (SA) within the maltreated children compared to the controls. All results were corrected for multiple comparisons using a monte-carlo null-*z* simulation (<.05) and adjusted for interhemispheric comparison. The *colour bar* visualises the log10 significance value of the clusters (4/−4 = *p* < .0001)

We first investigated the main effect of maltreatment exposure on cortical thickness across each hemisphere to identify regions associated with maltreatment including age, sex, IQ, and ICV as covariates of no interest. The maltreated group was found to have significantly reduced cortical thickness within one frontal cluster in the right hemisphere, compared to the non-maltreated group. The frontal cluster’s peak coordinate (Fig. 1; Table 2, cluster 1: *x* = 8, *y* = 37, *z* = −4; cluster corrected *p* < .05) fell within the medial orbitofrontal cortex, with the cluster extending into aspects of the anterior cingulate and superior frontal gyrus. No other clusters were found to be significantly thinner or thicker within the maltreated group compared to the non-maltreated peers.

**Table 2:** Significant clusters of surface-based measure group differences between the maltreated and non-maltreated group

	Cluster no.	L/R	Max-log_10_	*p*_cluster_	Area (mm^2^)	Local maxima (*x*, *y*, *z*)		
Cortical thickness								
Control > maltreated								
Rostral ACC/superior frontal	1	R	3.95	.002*	2429	8	37	−4
Local gyrification								
Control < maltreated								
Superior parietal	3	L	−2.57	.017*	585	−25	−58	53

	Cluster no.	L/R	Controls		Maltreated		*F*	*p*
			Mean	SD	Mean	SD		
Surface area								
Control > maltreated								
Inferior parietal	2	R	5951	703.92	5573.77	704.40	11.99	>.01**

*L* left, *R* right, *p*_*cluster*_ cluster probability

* Corrected for multiple comparisons using a monte-carlo null-*z* simulation and adjusted for interhemispheric comparison

** Survived false discovery rate correction (*q* < .05) and adjusted for interhemispheric comparison

#### Surface area

Independent *t* tests, corrected for multiple comparisons per hemisphere (Benjamini and Hochberg 1995), were performed on each gyral region (34 gyral regions) per hemisphere with age, sex, IQ, and ICV included as covariates of no interest. The right inferior parietal cortex had a significantly reduced surface area in the maltreated group compared to control non-maltreated subjects (Fig. 1; Table 2, cluster 2: *F* = 11.99, *p* < .002; FDR corrected for multiple comparisons and hemispheres). Notably, bilateral entorhinal cortex [left, *F*(1,115) = 4.65, *p* = .03; right, *F*(1,115) = 4.62, *p* = .03], bilateral supramarginal [left, *F*(1,115) = 5.76, *p* = .02; right, *F*(1,115) = 6.03; *p* = .02] and right middle temporal cortex [*F*(1,115) = 4.7, *p* = .03] were found to show a reductions in surface area, however, these did not survive corrections for multiple comparisons. Reductions in SA of the left lingual gyrus and left middle temporal area (areas of reduced SA reported in Kelly et al. (2013) did not reach statistical significance—although the direction of the effect was consistent with our previous study [left lingual, *F*(1,115) = 3.30; *p* = .07; left middle temporal, *F*(1,115) = 3.22; *p* = .07]. No other regions were found to survive correction for multiple comparisons.

#### Local gyrification

Investigating regions of atypical local gyrification associated with maltreatment with age, sex, IQ, and ICV included as covariates of no interest, one significant cluster was identified within the left hemisphere reflecting increased lGI within the maltreated group compared to the non-maltreated group (Fig. 1; Table 2, cluster 3: *x* = −25, *y* = −58, *z* = 53; Monte Carlo null-*z* simulation corrected *p* < .05). The peak was located within the superior parietal cortex.

### The influence of sex on the impact of maltreatment on GMV

#### Cortical thickness

In the analysis of cortical thickness, with age, IQ, and ICV included as covariates of no interest, there was no significant main effect of sex [*F*(1,115) = 2.56, *p* = .11] and sex and group did not significantly interact within the cluster encompassing the medial OFC/anterior cingulate cortex/superior frontal cortex [*F*(1,115) = .19, *p* = .67], suggesting no differential effect of maltreatment exposure on cortical thickness in male and female children in this region. In the second step of the analyses, no interactions between sex and maltreatment were detected for cortical thickness in either the left or right hemisphere.

#### Surface area

In the analysis of surface area, with age, IQ, and ICV included as covariates of no interest, there was no significant main effect of sex [*F*(1,115) = .29, *p* = .59] and no group by sex interaction [*F*(1,115) = .12, *p* = .73] within the inferior parietal cortex, indicating that the atypical surface area within this region associated with maltreatment was similar in both males and females. The analysis was run excluding age, IQ and ICV as covariates and while there was no group by sex interaction [*F*(1,118) = .05, *p* = .83], there was a significant main effect of sex [*F*(1,118) = 9.7, *p* < .05]. This is unsurprising given that global surface area was found to be significantly different between the sexes.

In the second step of the analyses, a significant interaction between group and sex was observed in bilateral regions of insula [left, *F*(1,115) = 9.02, *p* < .01; right, *F*(1,115) = 8.23, *p* = .01] and pars triangularis [right, *F*(1,115) = 6.70, *p* = .01]. However, these regions did not survive cluster correction for multiple comparisons. No other regions were found to show a main effect of sex or a group by sex interaction.

#### Local gyrification

In the analysis local gyrification, with age, IQ, and ICV included as covariates of no interest, there was no significant main effect of sex [*F*(1,115) = 3.41, *p* = .07] and no group by sex interaction [*F*(1,115) = .01, *p* = .91], indicating that the atypical local gyrification in parietal cortex associated, which characterised the maltreated group overall, was similar in both sexes.

**Fig. 2:**
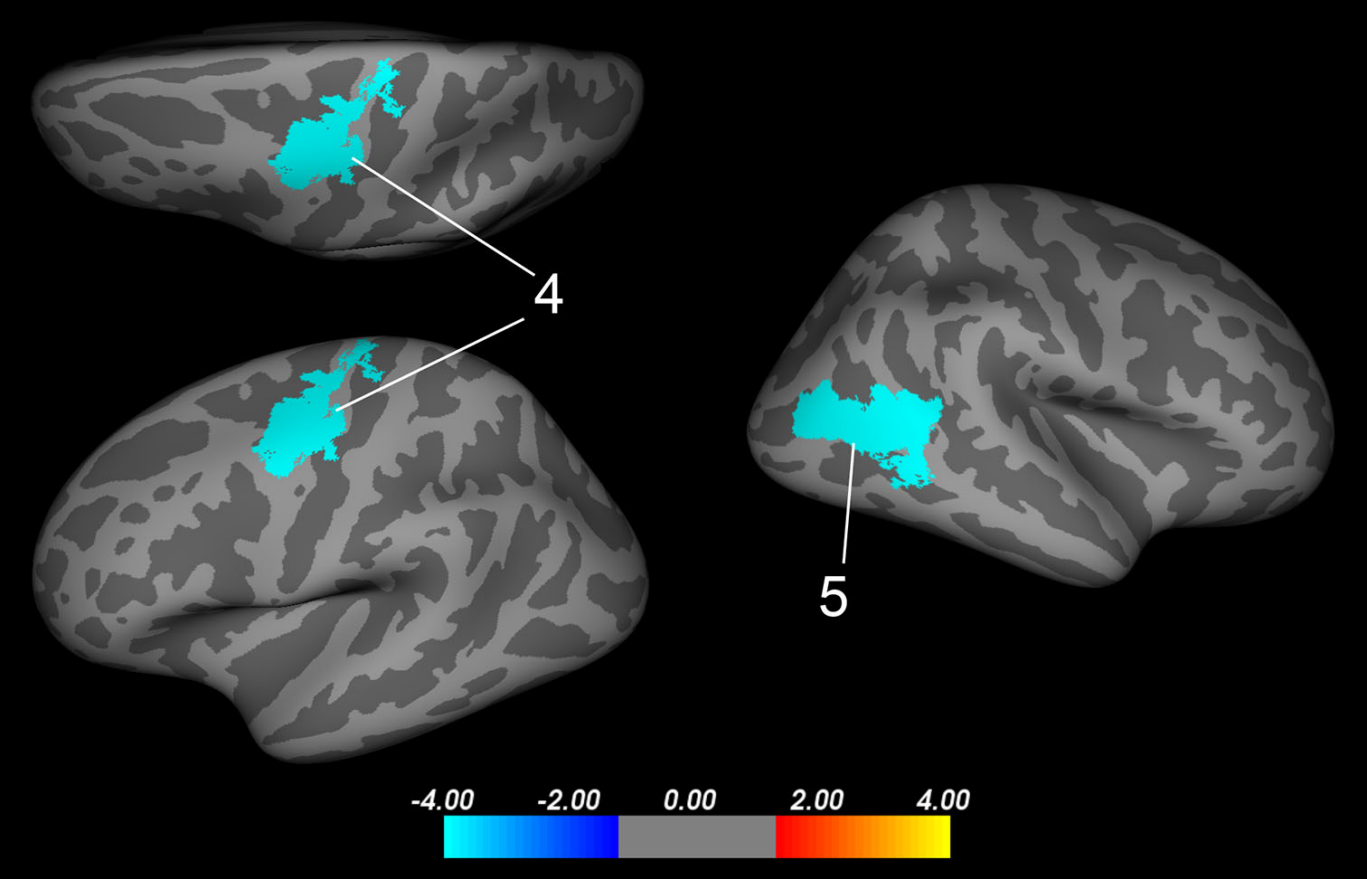
Clusters of a significant interaction between maltreatment exposure and sex in local gyrification index. Significant clusters reflecting group by sex interaction in local gyrification across both hemispheres projected onto an inflated average surface. In the left hemisphere, cluster *4* lies within the precentral gyrus (*x* = −37, *y* = −6, *z* = 45). In the right hemisphere, cluster *5*’s peak lies within the middle temporal lobe and extends into aspects of the inferior parietal gyrus (*x* = 44, *y* = −68, *z* = 7). *LH* left hemisphere; *RH* right hemisphere. All results were corrected for multiple comparisons using a Monte-Carlo null-*z* simulation (<.05) and adjusted for interhemispheric comparison. The colour bar visualises the log10 significance value of the clusters (4/−4 = *p* < .0001)

**Fig. 3:**
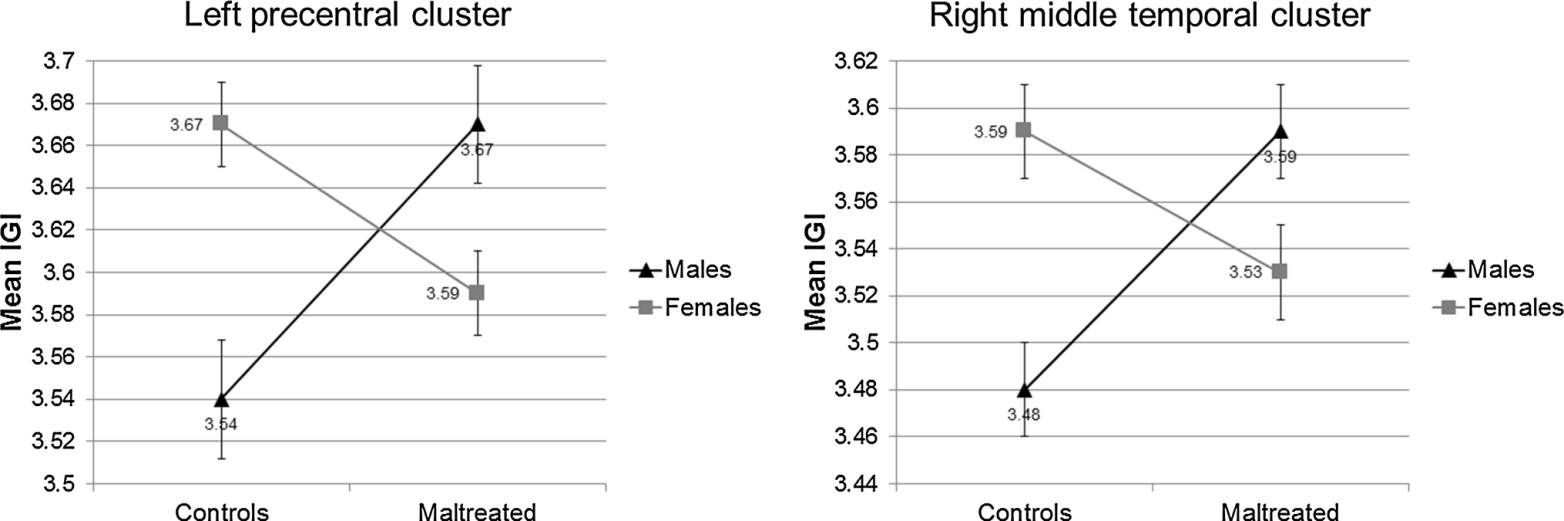
Plot of the mean local gyrification values extracted from the left precentral (cluster *4*, Fig. 2) and right middle temporal/inferior parietal (cluster *5*, Fig. 2) clusters split by group and sex

In the second step of the analyses, two significant bilateral clusters were revealed, indicating an interaction between sex and maltreatment. The peak coordinate within the left hemisphere cluster was located within the precentral gyrus (Fig. 2; Table 3, cluster 4: *x* = −34, *y* = −6, *z* = 45; cluster corrected *p* < .05). Within the right hemisphere a significant cluster was identified with its peak located within middle temporal gyrus (Figs. 2, 3, cluster 5: *x* = 44, *y* = −68, *z* = 7; cluster corrected *p* < .05) and extending into aspects of the inferior parietal cortex (Fig. 2, cluster 5). Inspection of the extracted adjusted mean lGI indicated a similar pattern across both significant clusters: a reduction in lGI in maltreated females compared with non-maltreated females and an increase in lGI in maltreated males compared with non-maltreated males (Fig. 3). However, follow-up pairwise comparisons of the adjusted means demonstrated that the maltreated males differed significantly from their male control counterparts and also the male and female controls differed significantly. The maltreated males and females did not differ significantly in lGI within both of the clusters.

**Table 3:** Significant clusters of interaction between maltreatment exposure and sex in local gyrification index

Anatomical regions	Cluster no.	L/R	Max-log_10_	*p*_cluster_	Area (mm^2^)	Local maxima (*x*, *y*, *z*)		
precentral gyrus	4	L	−4.00	<.002*	1402	−34	−6	45
Middle temporal/inferior parietal	5	R	−4.00	<.002*	1509	44	−68	7

*L* left, *R* right, *p*_*cluster*_ cluster probability

* Corrected for multiple comparisons using a monte-carlo null-*z* simulation and adjusted for interhemispheric comparison

For all analyses excluding our set of covariate (age, sex, IQ, and ICV) did not significantly alter the pattern of findings. Furthermore, entering pubertal status as a covariate of no interest also did not significantly change the pattern or significance of the results.

### Psychiatric symptomatology and maltreatment severity

A 2 × 2 ANOVA was employed to explore group by sex interactions in psychiatric symptomatology and maltreatment severity. We found that there was no main effect of sex and no group by sex interaction for any of the subscales or total scores on the TSCC or SDQ. No significant associations were found between the extracted mean cortical values from the significant clusters and maltreatment severity (as indexed by either the mean total Kaufman score or maltreatment subtype scores) within the maltreated group.

## Discussion

The current study sought to systematically investigate regional differences in CT, SA and lGI associated with maltreatment and to subsequently investigate possible sex differences in how maltreatment may impact these indices. We found that maltreatment exposure was associated with a decrease in CT within a frontal cluster including the medial OFC and anterior cingulate, a SA decrease within the inferior parietal cortex and an lGI increase within the superior parietal cortex. Only in our measure of local gyrification did we find an interaction between maltreatment and sex. Specifically, two clusters within the left precentral cortex and right tempo-parietal junction showed increased lGI in the male children who had been exposed to maltreatment relative to their peers, but this pattern was not observed for female children.

### The influence of maltreatment on surface-based measures of cortical structure

#### Cortical thickness

Reduced CT was observed within a region of medial OFC, anterior cingulate and superior frontal cortex, similar to a cluster of reduced CT reported in our previous study, which included a subsample of the current cohort of participants. This suggests that CT decreases in this frontal region may be a reliable correlate of maltreatment experience, potentially acting as a precursor to volumetric deficits seen in adult samples (Cohen et al. 2006; Kelly et al. 2013; Kitayama et al. 2006; Lim et al. 2014). Importantly, animal studies of early stress, and volumetric and surface-based studies of individuals with PTSD have indicated the structural sensitivity of the frontal and prefrontal regions to stress (Arnsten 2009; Bremner 1999; Dickie et al. 2013; Geuze et al. 2008; Kitayama et al. 2006; Shin et al. 2006). Atypical structure within this region in PTSD samples, and the association between these areas and emotion regulation (Etkin et al. 2011), suggests that these cortical thickness differences may be relevant for our understanding of the relationship between environmental adversity and maladaptive emotional regulation. Due to the cubic trajectory of cortical thickness development within frontal regions across adolescence (Shaw et al. 2008), it will be important to collect longitudinal data in maltreated samples. As cortical thickness decreases are believed to optimise computation in frequently used circuits through processes, such as apoptosis (Sowell et al. 2007), one possibility is that maltreated individuals recruit these frontal regions to a greater degree than their non-maltreated peers.

#### Surface area

Reduced SA was detected within the parcellated region of the right inferior parietal cortex within the maltreated males and females compared to their non-maltreated peers; by contrast, our previous study found SA decreases in left middle temporal and lingual gyri associated with maltreatment (Kelly et al. 2013). It is not possible to arbitrate where the differences in the SA findings stem from. These could reflect differences in maltreatment subtype experience across the studies (the proportion of maltreated children reporting experiences of physical abuse was much higher in the Kelly et al. (2013) study), but much larger samples will be required to determine specific effects of different maltreatment types—especially as poly-victimisation is the norm, rather than the exception in these samples (Turner et al. 2010). In addition, it is perfectly possible that there is natural variability across SA in these regions in typically developing participants, which might equally account for this inconsistency. Currently there is insufficient normative data to draw reliable conclusions in this regard. The inferior parietal cortex is associated with the detection of facial emotional stimuli (Adolphs et al. 1996, 2000; Sarkheil et al. 2013), known to be atypical in maltreated participants (Pollak et al. 2001; Pollak and Sinha 2002). It is also noteworthy that volumetric differences within the parietal cortex have been observed in adults with childhood histories of maltreatment, who also present with concurrent psychiatric disorder (Bremner et al. 2005; Irle et al. 2005).

#### Local gyrification

Surprisingly, we observed an increase in lGI within the superior parietal cortex, in contrast to the findings of Kelly et al. (2013) which reported maltreatment related decreases in lGI in the insula and lingual gyrus (Kelly et al. 2013). The reason for the different pattern of findings across studies is not clear. As we highlighted in relation to the SA findings, it is not possible to arbitrate where the differences in the findings between the two studies stem from. These could reflect differences in maltreatment subtype experiences across the studies, but much larger samples will be required to examine this question. As we also discussed above, variability in typically developing participants may also account for inconsistency across the studies, but more normative data is required to investigate this possibility.

One previous study has reported GMV alterations in the superior parietal cortex in children exposed to maltreatment (Edmiston et al. 2011). More broadly, lGI differences in the superior parietal cortex have been reported in adolescents with conduct disorder—a common outcome associated with childhood maltreatment experience (Fairchild et al. 2015; Hyatt et al. 2012).

### Sex differences, maltreatment and surface based indices

Our investigation of the potential interaction of sex and maltreatment exposure at a whole brain level identified two significant regions, the left precentral gyrus and the right tempo-parietal junction. Both clusters displayed a similar pattern, such that the control females had greater lGI in these regions compared to males, while an inverse relationship between males and females was observed in the maltreated sample. However, only maltreated males were found to significantly differ from the control males, and while maltreated females did show a trend for significantly different lGI from both control females and maltreated males, this difference did not reach significance. Gradual and linear decreases in lGI are observed across childhood and adolescence (Klein et al. 2014; Su et al. 2013), with the strongest reductions in precentral, temporal and frontal regions which may suggest that observable increases in lGI within the maltreated males are indicative of a delayed maturational trajectory. As alterations in gyrification has been linked to connectivity patterns (Van Essen 1997), maltreated males male show different profiles of connectivity to their non-maltreated counterparts. However, longitudinal studies and specific connectivity analyses would be crucial in providing support for such a hypothesis. This suggests that maltreatment experience has a more pronounced impact on lGI within these regions in boys compared to girls.

The findings of an interaction between sex and maltreatment exposure within the precentral cortex is of interest given the role of this region in the visual recognition of emotion (Adolphs et al. 2000, 2003; Pitcher et al. 2008). Sex differences in functional activation in this region during emotional processing and the cognitive control of emotional states have been observed in normative samples (Domes et al. 2010; Stevens and Hamann 2012) and in patients with major depression (Frodl et al. 2009). The right hemisphere cluster was in the middle temporal and inferior parietal cortex, commonly referred to as the tempo-parietal junction. This region has been consistently shown to be functionally activated during the selective attribution of mental states and theory of mind (Saxe and Kanwisher 2003; Saxe and Wexler 2005), and sex differences within these domains are found in normative samples (Baron-Cohen 2000; Brown et al. 1996). Atypical theory of mind and concurrent structural deficits within the tempo-parietal region are reported in a range of psychiatric disorders associated with maltreatment (Hezel and McNally 2014; Huprich et al. 2012; Jaworska et al. 2014; Nesvåg et al. 2014; Palaniyappan and Liddle 2012; Shestyuk and Deldin 2014).

Such differences in the precentral and tempo-parietal regions may represent possible neural substrates implicated in the differential psychiatric risk trajectories seen in maltreated males and females (Bos et al. 2011; Keyes et al. 2012). However, it is important to remain cautious given that sex differences in cortical structure and function do not necessarily reflect isomorphic behavioural differences (De Vries 2004; De Vries and Södersten 2009; Piefke et al. 2005). One possibility is that these structural differences are predictive of future psychiatric presentation; it will therefore be important to follow up such samples longitudinally.

## Limitations

The present findings should be interpreted in light of a number of limitations. The use of a cross-sectional design prohibits the ability to make causal inferences about the experience of childhood maltreatment and the observed atypical cortical structure within the males and females of the maltreated group, which a longitudinal design would help to determine (Shaw et al. 2013). Furthermore, due to systematic differences in the pre-processing and analysis techniques of VBM and FreeSurfer, systematic comparison between the current findings and those of our previous investigation into sex differences in the impact of maltreatment on GMV is problematic (e.g. Blankstein et al. (2009)) without an appropriate system for integration between the methods (Makris et al. 2006). In addition, although we controlled our covariates (age, IQ, sex, SES, pubertal status and ICV), a possibility remains that these factors may impact cortical structure in a non-linear manner; further investigations into the interaction between these variables and maltreatment would help to shed light on this possibility. Key strengths of the current study include a non-clinical community sample with independently documented experiences of maltreatment, and the careful matching on a range of socio-demographic variables between a large non-psychiatric sample of maltreated children and non-maltreated peers, which increases the likelihood that any observed differences in cortical structure are due to maltreatment experience.

## Conclusions

The current study sought to investigate regional differences in cortical thickness, surface area and local gyrification associated with maltreatment and to subsequently investigate possible sex differences in how maltreatment may impact these indices. While sex differences were observed in relation to lGI, the impact of maltreatment across the surface-based measures was largely similar between sexes.

The sample of maltreated males and females displayed decreased CT within a region of the medial OFC, anterior cingulate, and superior frontal gyrus, consistent with previous findings (Kelly et al. 2013), suggesting that such alterations may be a reliable correlate of maltreatment experience. SA and lGI differences in the maltreated group compared to the non-maltreated group were found in right inferior parietal and left superior parietal regions, respectively.

Sex differences were found only within two clusters of lGI, left precentral gyrus and right tempo-parietal junction; in these regions an interaction between maltreatment exposure and sex was observed. Differences in lGI were only observed in maltreated males compared to their control counterparts, suggesting a potentially greater sensitivity in males to such structural changes following maltreatment experience. These regions have been implicated in emotion regulation and theory of mind processes, respectively, both commonly found to be affected in maltreated populations (Rogosch et al. 1995). It is possible that sex differences in lGI may be associated with psychiatric risk trajectories apparent in in maltreated males and females (Keyes et al. 2012; Mesman et al. 2001). Future longitudinal studies are required to determine the functional significance of these cortical differences and whether they represent markers of latent vulnerability in relation to future psychiatric outcome.

## Electronic supplementary material

Below is the link to the electronic supplementary material.

Supplementary material 1 (DOCX 15 kb)
