# Supplementary material for: The sexually dimorphic impact of maltreatment on cortical thickness, surface area and gyrification
Source: J Neural Transm (Vienna). 2016 Feb 27;123(9):1069–83. doi: 10.1007/s00702-016-1523-8 (PMC5003912; doi:10.1007/s00702-016-1523-8)
Supplement: Supplementary file 1 — Supplementary material 1 (DOCX 15 kb) [file 702_2016_1523_MOESM1_ESM.docx]

# Supplemental material: The sexually dimorphic impact of maltreatment on cortical thickness, surface area and gyrification

Supplemental table S1.2. Global measurements of estimated total intracranial volume, and surface-based measures of cortical structure between the maltreated and non-maltreated groups.

| **By group** |  |  |  |  |  |  |
| --- | --- | --- | --- | --- | --- | --- |
|  | *Control (n = 60)* | |  | *MT (n = 62)* | | *p* |
|  | *Mean* | *SD* |  | *Mean* | *SD* |  |
| Estimated ICV | 1479214.0 | 122189.4 |  | 1446047.6 | 117034.3 | 0.13 |
| Mean CT | 2.65 | 0.09 |  | 2.65 | 0.10 | 0.86 |
| Mean lGI | 3.18 | 0.12 |  | 3.17 | 0.12 | 0.94 |
| Total SA | 173694 | 15367 |  | 169952 | 13780 | 0.16 |
|  |  |  |  |  |  |  |
| **By sex** |  |  |  |  |  |  |
|  | *Males (n = 37)* | |  | *Females (n = 47)* | | *p* |
|  | *Mean* | *SD* |  | *Mean* | *SD* |  |
| Estimated ICV | 1512992.0 | 115087.5 |  | 1416473.0 | 106273.4 | <0.001 |
| Mean CT | 2.65 | 0.10 |  | 2.65 | 0.10 | 0.86 |
| Mean lGI | 3.21 | 0.11 |  | 3.15 | 0.13 | 0.01 |
| Mean SA | 177477 | 13271 |  | 166640 | 13990 | <0.001 |
| ICV: Intracranial volume; CT: cortical thickness; lGI: local gyrification index; All values were computed across both hemispheres. All p values derived from t-tests. | | | | | | |
